# The structure of the Moco carrier protein from *Rippkaea orientalis*

**DOI:** 10.1107/S2053230X20011073

**Published:** 2020-08-28

**Authors:** Joern Krausze, Thomas W. Hercher, Archna Archna, Tobias Kruse

**Affiliations:** a Institute of Plant Biology, TU Braunschweig, 38106 Braunschweig, Germany

**Keywords:** molybdenum cofactor, Moco carrier protein, Rossmann fold, molecular docking

## Abstract

The molybdenum cofactor (Moco) is the prosthetic group of all molybdenum-dependent enzymes except for nitrogenase. The multistep biosynthesis pathway of Moco and its function in molybdenum-dependent enzymes are already well understood. The mechanisms of Moco transfer, storage and insertion, on the other hand, are not. In the cell, Moco is usually not found in its free form and remains bound to proteins because of its sensitivity to oxidation. The green alga *Chlamydomonas reinhardtii* harbors a Moco carrier protein (MCP) that binds and protects Moco but is devoid of enzymatic function. It has been speculated that this MCP acts as a means of Moco storage and transport. Here, the search for potential MCPs has been extended to the prokaryotes, and many MCPs were found in cyanobacteria. A putative MCP from *Rippkaea orientalis* (RoMCP) was selected for recombinant production, crystallization and structure determination. RoMCP has a Rossmann-fold topology that is characteristic of nucleotide-binding proteins and a homotetrameric quaternary structure similar to that of the MCP from *C. reinhardtii*. In each protomer, a positively charged crevice was identified that accommodates up to three chloride ions, hinting at a potential Moco-binding site. Computational docking experiments supported this notion and gave an impression of the RoMCP–Moco complex.

## Abstract 2

The structure of a Moco carrier protein from *Rippkaea orientalis* is presented together with evidence for its selective binding of the mononucleotide variety of molybdenum cofactor.

## 1. Introduction   

**Figure 1:**
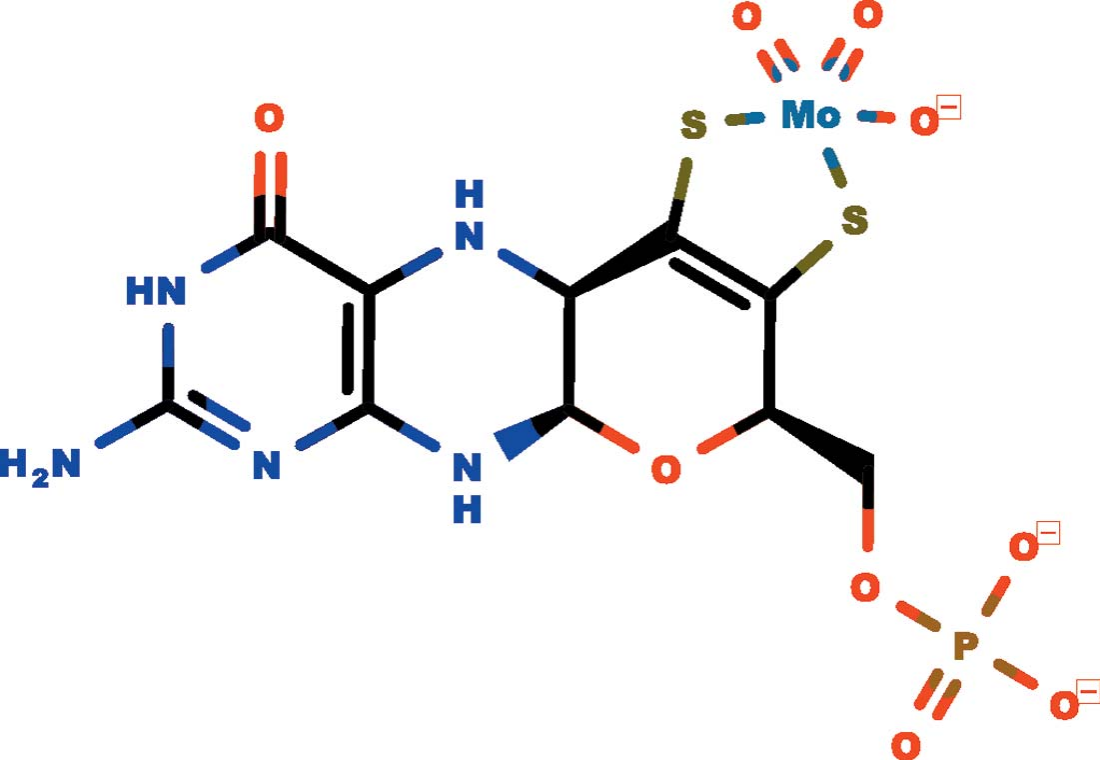
The molybdenum cofactor.

Molybdenum is an essential trace element in most species (Mendel, 2013 ▸). The abundant chemical form of molybdenum in the environment is the biochemically inactive oxyanion molybdate, which gains activity when complexed by the dithiolene motif of a specific tricyclic pterin called molybdo­pterin (MPT). The compound thus formed, named the molybdenum cofactor (Moco; Fig. 1 ▸), is then able to operate as a prosthetic group of Moco-dependent enzymes (Mo-enzymes). The chemical composition of Moco was first determined by Rajagopalan & Johnson (1992 ▸). The corresponding chemical structure initially remained elusive, as Moco is not amenable to the procedures used in the isolation of natural products, and was finally solved as part of the aldehyde oxidoreductase holoenzyme complex from *Desulfovibrio gigas* (Romão *et al.*, 1995 ▸). Since then, researchers have identified and structurally elucidated a large number of Mo-enzymes (Hille *et al.*, 2014 ▸) and have made considerable progress towards understanding the Moco-biosynthesis pathway.

Moco originates from one guanosine triphosphate (GTP) molecule through a multi-step biosynthesis pathway common to Moco-utilizing organisms throughout all three domains of life (Mendel & Leimkühler, 2015 ▸). The majority of bacterial Mo-enzymes, however, use the molybdopterin guanine di­nucleotide (MGD) or the molybdopterin cytosine dinucleotide (MCD) derivative of Moco (Hille *et al.*, 2014 ▸; Mendel & Leimkühler, 2015 ▸). In contrast, these derivatives do not exist in eukaryotes.

Moco carrier proteins (MCPs) neither belong to the Moco-biosynthesis pathway nor qualify as Mo-enzymes as they are non-enzymatic proteins within molybdenum metabolism. MCPs were initially speculated to exist after protein fractions from the cell lysates of certain species showed the ability to reconstitute the activity of Moco-free *Neurospora crassa* nitrate reductase (NR) even though these fractions had no Mo-enzyme activity of their own (Amy & Rajagopalan, 1979 ▸; Aguilar *et al.*, 1991 ▸). The first succeessful isolation of an MCP was that from the green alga *Chlamydomonas reinhardtii* (CrMCP; Ataya *et al.*, 2003 ▸). CrMCP binds Moco and significantly extends the half-life of Moco under oxic *in vitro* conditions (Fischer *et al.*, 2006 ▸). In the cytosol of *C. reinhardtii*, free Moco is not available for transfer to Mo-enzymes (Aguilar *et al.*, 1992 ▸), and hence a sudden increase in demand for Moco by the Mo-enzymes cannot be satisfied by this source. These facts suggest a triple role for CrMCP in (i) protecting Moco from oxidative degradation, (ii) stockpiling Moco for a sudden increase in Mo-enzyme expression and (iii) shuttling the product of Moco biosynthesis from source to sink. In higher plants, MCPs as such do not exist, but Moco-binding proteins fulfill a similar function (Kruse *et al.*, 2010 ▸). Structurally, MCPs belong to the Pfam family of lysine decarboxylases. The structure of CrMCP revealed that MCPs have a Rossmann-like topology that is common among nucleotide-binding proteins, which is in line with the notion of Moco being a nucleotide, as it solely derives from one GTP molecule. A positively charged depression on the surface of CrMCP can potentially accommodate Moco and compensate for its negatively charged groups. *In silico* docking experiments support this notion (Fischer *et al.*, 2006 ▸). The CrMCP–Moco complex, however, has so far resisted crystallization.

In bacteria, on the other hand, little is known about the prevalence and role of MCPs. However, the situation is more complicated than in eukaryotes because of the presence of MGD and MCD. In this work, we provide biochemical evidence that MCPs exist in bacteria, discriminate Moco from MGD and MCD, and transfer Moco to apo NR and reconstitute NR activity. We further present the structure of an MCP from the cyanobacterium *Rippkaea orientalis* at near-atomic resolution together with *in silico* docking studies that pinpoint the putative Moco-binding site.

## 2. Methods   

### 2.1. Cloning, expression and purification of *R. orientalis* MCP   

We ordered a synthetic *R. orientalis mcp* gene (GenBank accession No. WP_012595913) with codon usage optimized for *Escherichia coli* from MWG Eurofins. The gene arrived in a pEX-A2 plasmid and served as a template for PCR amplification. The BamHI and HindIII restriction sites intrinsic to the PCR primers (shown in capitals in Table 1 ▸) allowed the generation of a DNA fragment with sticky ends. The fragment was subsequently ligated into derivatives of the pQE80 expression vector, which enabled us to produce Hexa-His-RoMCP and Twin-Strep-RoMCP constructs (Ringel *et al.*, 2013 ▸, 2015 ▸). While the former was used for crystallization and structure determination, the latter was used for the quantification of bound Moco. For expression, we transformed either of the constructs into *E. coli* strain BL21 and cultivated the bacteria in LB medium in the presence of 50 mg l^−1^ ampicillin. Expression was induced with 0.1 *M* isopropyl β-d-1-thio­galactopyranoside when the culture reached an optical density at λ = 600 nm of 0.5. Expression proceeded for 20 h at 20°C. After harvesting, the cells and the RoMCP within them were further processed using buffers *L* [0.2 *M* Tris–HCl pH 8.0, 0.3 *M* NaCl, 2%(*v*/*v*) glycerol], *W* [0.1 *M* Tris–HCl pH 8.0, 0.5 *M* NaCl, 2%(*v*/*v*) glycerol] and *C* [0.025 *M* Tris–HCl pH 8.0, 0.3 *M* NaCl, 2%(*v*/*v*) glycerol]. Firstly, the cells were resuspended in buffer *L* (Twin-Strep-RoMCP) or buffer *L* plus 0.01 *M* imidazole (Hexa-His-RoMCP) and subjected to cell lysis using a French pressure cell at 100 MPa. After centrifugation at 50 000*g*, the cleared cell lysate was applied onto a Strep-Tactin II column (Twin-Strep-RoMCP) or a HisTrap column (Hexa-His-RoMCP). The column was then rinsed with buffer *W* (Twin-Strep-RoMCP) or buffer *W* plus 0.02 *M* imidazole (Hexa-His-RoMCP). The protein was subsequently eluted with buffer *L* plus 0.025 *M* desthiobiotin (Twin-Strep-RoMCP) or buffer *L* plus 0.2 *M* imidazole (Hexa-His-RoMCP). Twin-Strep-RoMCP was subsequently used for the quantification of Moco and the reconstitution assay. In contrast, Hexa-His-RoMCP was subjected to gel filtration with a Superdex 10/300 Increase gel-filtration column to replace the current buffer system with buffer *C*. The fractions containing protein were pooled and concentrated to about 22.5 g l^−1^. Protein concentrations were determined by UV spectroscopy at λ = 280 nm using theoretical extinction coefficients for the RoMCP constructs of ∊_280_ = 23 490 *M* ^−1^ cm^−1^ for Twin-Strep-RoMCP and ∊_280_ = 12 490 *M* ^−1^ cm^−1^ for Hexa-His-RoMCP. In the case where more precise protein quantification was required, an assay based on bicinchoninic acid (BCA Protein Assay Kit, Thermo Fisher) was used to measure protein concentrations.

### 2.2. Quantification of Moco bound to RoMCP   

The quantification of the Moco content in RoMCP samples followed a previously published protocol (Hercher *et al.*, 2020 ▸). Briefly, the Moco content of a sample containing 100 pmol RoMCP was oxidized with a solution containing 1% I_2_ and 2% KI under acidic conditions and converted to the compound Form A. Form A was then deprived of its phosphate group by adding alkaline phosphatase, and the resulting dephospho-Form A was separated in an HPLC setup using a ReproSil-Pur Basic C-18 HD column with dimensions of 250 × 4.6 mm and 5 µm bead size and was measured with a fluorescence detector. From the area under the curve of the elution peak, the amount of dephospho-Form A in the sample was quantified, with a synthetic Form A standard (Klewe *et al.*, 2017 ▸) serving as a reference. The saturation of RoMCP with Moco was calculated as the ratio of the amount of measured dephospho-Form A to the amount of protein subjected to oxidation. The control sample for the Form A assay was the cytochrome *c* reductase (C*c*r) fragment from *N. crassa* NR, which is known not to bind Moco. The control sample was expressed and purified identically to RoMCP.

### 2.3. Reconstitution assay and NR activity assay   

The capability of RoMCP to transfer Moco to apo NR was determined in a reconstitution assay using an extract of a Moco-free *N. crassa nit-1* mutant (Nason *et al.*, 1970 ▸, 1971 ▸). The reconstitution started by supplementing a 20 µl volume of *nit-1* extract with 5 µl buffer *A* (50 m*M* Na_2_HPO_4_/NaH_2_PO_4_ pH 7.2, 200 m*M* NaCl, 5 m*M* ethylenediaminetetraacetic acid, 30 m*M* reduced glutathione, 2 µl glycerol, 5 m*M* Na_2_MoO_4_) and 5 µl test sample containing RoMCP followed by incubation overnight on ice. In a separate sample, 1 U phosphodiesterase (PDE) was added to the mixture to initiate the transfer of potentially bound MGD or MCD. The control sample for the reconstitution was again the C*c*r fragment. The reconstituted NR activity was then measured in an activity assay that colorimetrically monitored the reductive formation of nitrite. To this end, buffer *A* minus glutathione was supplemented with 20 m*M* KNO_3_, 2 m*M* NADPH and 0.1 m*M* FAD. An equal volume of the resulting mixture was added to each of the overnight samples and incubated at room temperature in the dark to allow the reconstituted NR to convert nitrate to nitrite. The reaction was stopped after 30 min by adding 40 µl of a 600 m*M* zinc acetate solution. Afterwards, 400 µl of a freshly prepared solution consisting of 0.5% sulfonamide and 0.01% *N*-(1-naphthyl)ethylenediamine in 1.5 *M* HCl was added. In the presence of nitrite anions, sulfanilamide and *N*-(1-naphthyl)ethylenediamine form a bright pink azo dye, which can be quantified by UV–Vis spectroscopy at λ = 540 nm.

For the reconstitution assay, we tested the significance of differences in the means of two data groups using an unpaired, two-tailed Student’s *t*-test (Gosset, 1908 ▸). The corresponding *p*-value represents the likelihood that the measured difference is the result of random chance. The test statistics were assumed to follow a Gaussian distribution.

### 2.4. Crystallization of RoMCP   

We used several commercial screens for the initial crystallization screening, including the MPD Suite from Qiagen. Crystals suitable for diffraction experiments with no need for further refinement grew from condition No. 56 of the MPD Suite (Table 2 ▸). The mother liquor provided sufficient cryoprotection and the crystals could be immediately flash-cooled for data collection.

### 2.5. Data collection and processing   

Data collection was carried out on beamline X06DA (Bingel-Erlenmeyer *et al.*, 2011 ▸) operated by the Paul Scherrer Institute at the Swiss Light Source (SLS), Villigen, Switzerland. Data were recorded using a PILATUS 2M-F detector in single photon-counting mode. Diffraction images were indexed and Bragg reflections were integrated with *XDS* (Kabsch, 2010 ▸). The intensities were merged and averaged and the most likely space group was determined with *AIMLESS* (Evans, 2006 ▸, 2011 ▸; Evans & Murshudov, 2013 ▸). Initially, the high-resolution cutoff was chosen conservatively according to the *I*/σ(*I*) threshold of 2. Later, after the building of the structure was almost complete, the high-resolution cutoff was reassessed through paired refinement (Karplus & Diederichs, 2012 ▸) and the diffraction data were reprocessed accordingly. Table 3 ▸ displays the complete data-collection and processing statistics.

### 2.6. Structure determination and refinement   

The phase problem was solved by molecular replacement with *Phaser* (McCoy *et al.*, 2007 ▸) using a single chain of the CrMCP structure (PDB entry 2iz6; Fischer *et al.*, 2006 ▸) as a search model. The placed search model was subsequently improved by a single run of *SHELXE* with 50 cycles of polyalanine autotracing to remove the model bias that severely affected the quality of the initial electron density (Sheldrick, 2008 ▸; Thorn & Sheldrick, 2013 ▸). The correct side chains were then manually added to the polyalanine model in *Coot* (Emsley *et al.*, 2010 ▸) and the structure was refined by iterative steps of refinement in *REFMAC*5 (Murshudov *et al.*, 2011 ▸) and manual building in *Coot*, while the progress of refinement was monitored through the *R* and *R*_free_ values. After no further improvement of the residuals could be achieved, the structure was subjected to paired refinement (Karplus & Diederichs, 2012 ▸) using the *PDB-REDO* server (Joosten *et al.*, 2014 ▸) against a data set comprising the full resolution range recorded on the detector. The new high-resolution limit determined through the paired refinement was then used in further building and refinement cycles with the optimized refinement parameters found by *PDB-REDO*, including anisotropic *B*-factor refinement and riding H atoms. The refinement process was stopped when the *R* and *R*_free_ values converged. The complete refinement statistics are shown in Table 4 ▸. The structure was deposited in the PDB as entry 6y01.

### 2.7. Small-angle X-ray scattering (SAXS)   

We conducted the SAXS experiments on beamline BM29 (Pernot *et al.*, 2013 ▸) of the European Synchrotron Radiation Facility, Grenoble, France by loading an RoMCP sample at a concentration of 38 µ*M* into a quartz capillary with an automated sample changer and exposing it to X-rays of 12 500 eV at a temperature of 293 K. The scattering intensities were recorded within a momentum transfer range of 3.2 × 10^−3^ to 5.0 × 10 ^−1^ Å^−1^ using a PILATUS 1M hybrid photon-counting detector over ten scattering images with an exposure time of 0.5 s per image. While being irradiated, the protein solution was flowing steadily in order to avoid radiation damage. The scattering data were processed using programs from the *ATSAS* suite (Franke *et al.*, 2017 ▸). Appraisal of the scattering curves revealed the influence of radiation damage at minimal scattering angles and a high noise level at large scattering angles. Therefore, we truncated the data set and removed data with a momentum transfer of below 2.7 × 10^−2^ Å^−1^ or above 4.5 × 10^−1^ Å^−1^ before we subjected the data to normalization, averaging and baseline correction through subtraction of the buffer scattering. We compared the theoretical scattering intensities for the tetramer, the *AB* dimer, the *AC* dimer and the monomer of RoMCP calculated from the crystal structure with the experimental intensities using *CRYSOL* (Svergun *et al.*, 1995 ▸). We also derived the distance distribution function from the experimental scattering intensities with *GNOM* (Svergun, 1992 ▸) and used it to calculate 20 low-resolution *ab initio* models with *DAMMIF* (Franke & Svergun, 2009 ▸). These models had a mean normalized spatial discrepancy 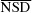 of 1.219 (88). A total of 19 models fell within 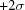 of 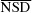 and were hence adequate for averaging and combining into a representative model of the protein envelope using *DAMAVER* and *SUPCOMB* (Kozin & Svergun, 2001 ▸; Volkov & Svergun, 2003 ▸).

### 2.8. Docking of Moco into RoMCP   

The genetic algorithm *GOLD* version 5.2.2 (Jones *et al.*, 1997 ▸) was used for the docking of Moco into RoMCP. The docking calculation used the Moco molecule extracted from the structure of *Pichia angusta* NR (PDB entry 2bih; Fischer *et al.*, 2005 ▸) as the ligand and the tetramer of RoMCP as the receptor, both of which were fully hydrogenated. Before docking, we modified the molybdenum center of Moco by replacing the Mo atom with a P atom as no proper parametrization of molybdenum was available. We assumed that phosphorus would be a satisfactory replacement as the resulting charge and geometry are similar to those of the molybdenum center of Moco. The putative binding pocket comprised all amino acids within a sphere of radius *r* = 15.0 Å with the origin at **o** = (−25.755, −22.473, 11.003) coinciding with the position of the C^ζ^ atom of Arg89 in protomer *A*. The radius of this sphere reached beyond the boundaries of protomer *A* and also included amino acids of protomers *B*, *C* and *D* to account for possible interprotomeric interactions of Moco. For the docking runs, the population size was 100 with a selection pressure of 1.1. The operator weights for crossover, mutation and migration were 90, 90 and 10, respectively. The ranking of the docking results was based on the GoldScore as implemented in *GOLD*.

### 2.9. Visualization of data   

We generated Fig. 3 with *ESPript* (Robert & Gouet, 2014 ▸) using a sequence alignment created by *Clustal Omega* (Madeira *et al.*, 2019 ▸) and the secondary structure from the PDB file of RoMCP as interpreted using *DSSP* (Kabsch & Sander, 1983 ▸). The topology diagram in Fig. 4(*a*) was drawn with *TopDraw* (Bond, 2003 ▸) and subsequently enhanced. We prepared the figures of three-dimensional protein structures using *UCSF ChimeraX* (Goddard *et al.*, 2018 ▸). *UCSF ChimeraX* uses the *MSMS* package (Sanner *et al.*, 1996 ▸) for the creation of solvent-excluded protein surfaces and *POV-Ray* (http://www.povray.org) for raytracing. The schematic of the protein–Moco interactions in Fig. 6 was created with *LigPlot*+ (Laskowski & Swindells, 2011 ▸). The SAXS envelope in Fig. 5 was made by converting the bead model generated by *DAMMIF* to a volumetric map using the *Situs* package (Wriggers, 2012 ▸).

## 3. Results and discussion   

### 3.1. Identification of a bacterial MCP   

A *BLASTP* search (Altschul *et al.*, 1990 ▸, 1997 ▸) against the NCBI database (NCBI Resource Coordinators, 2018 ▸) returned proteins homologous to CrMCP from all bacterial phyla. According to their automated annotations, these proteins belong to a variety of classes and families. Homologs from the phylum Cyanobacteria and phototrophic bacteria from the phylum α-Proteobacteria show the highest degree of sequence identity to CrMCP, in the range 50–60%. In contrast, homologs from the phyla Chlamydiae and ∊-Proteobacteria show the lowest degree of sequence identity to CrMCP, in the range 20–25%, and hence probably do not qualify as MCPs. This notion is in line with the fact that the members of the former two phyla are oxygen-evolvers and as such are in need of an MCP to protect the highly labile Moco (Witte *et al.*, 1998 ▸), while the members of the latter two phyla live in microaerophilic or anaerobic environments and are hence able to dispense with Moco protection through an MCP. Thus, we selected a target gene from a cyanobacterium for further study, increasing the likelihood of selecting a genuine MCP. We chose the MCP homolog from the organism *R. orientalis*, a cyanobacterium belonging to the genus *Rippkaea*, a recently established phylogenetic lineage formerly assigned to the genus Cyanothece (Mareš *et al.*, 2019 ▸). This putative MCP, referred to in the following as RoMCP, consists of 163 amino acids corresponding to a molecular weight of 17.1 kDa and shares 52.5% sequence identity with CrMCP. RoMCP comprises mostly negatively charged amino acids, as is reflected by its acidic pI of 5.29, which is close to the pI of 5.88 for CrMCP. After codon optimization, the *mcp* gene from *R. orientalis* could be expressed in *E. coli*, with yields of up to 5 µg of soluble protein per litre of culture.

### 3.2. Purification of RoMCP for crystallization   

**Figure 2:**
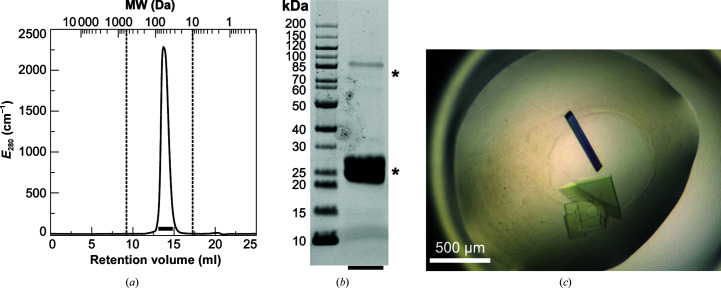
(*a*) Elution profile of RoMCP after passage through a Superdex 200 Increase 10/300 gel-filtration column. The retention volume of 13.7 ml corresponds to an estimated molecular weight of 60 kDa. The broken lines highlight the upper and lower exclusion limits of the column. The black bar indicates the fractions that were pooled and loaded onto an SDS–PAGE gel. (*b*) SDS–PAGE of purified RoMCP. Asterisks indicate the bands that were assigned to RoMCP and have apparent molecular weights of 20–25 and 80–85 kDa. (*c*) Monoclinic crytals of RoMCP.

After a two-step purification regimen involving HisTrap affinity purification and gel filtration, the RoMCP sample was pure, homogeneous and suitable for crystallization. The gel-filtration elution profile shows a single, symmetric peak, the retention volume of which indicates a molecular weight of about 60 kDa (Fig. 2 ▸ *a*). Considering the monomeric molecular weight of the construct of 18.9 Da, this alone would suggest the existence of an RoMCP trimer in solution. On the other hand, the fact that CrMCP forms a dimer of dimers (Fischer *et al.*, 2006 ▸) suggests a similar oligomeric state for RoMCP and renders a trimer unlikely, especially when considering that gel filtration is ill-suited for the precise determination of molecular weight. On denaturating SDS–PAGE, RoMCP shows a dominant band close to the monomeric molecular weight of the construct (Fig. 2 ▸ *b*). A band corresponding to a molecular weight of 80 to 85 Da was also visible, which we considered could be the consequence of a very stable tetramer that partially resists denaturation by SDS.

### 3.3. Binding of Moco and transfer to apo NR   

In an HPLC-based Form A assay, the measured saturation of RoMCP with Moco was 0.43 (4)% (*N* = 4). In contrast, the amount of Moco in the control sample was below the detection limit. The presence of Moco in the RoMCP sample and its absence from the control sample indicated that RoMCP is capable of binding Moco and hence is a genuine Moco carrier protein. Using expression conditions similar to ours, Fischer *et al.* (2006 ▸) reported no detectable saturation for CrMCP in a Form A assay. However, they were able to increase the saturation to 25% through optimal synchronization of intracellular protein and cofactor synthesis. For RoMCP, we were unable to improve the saturation with Moco by a similar means. However, the low occupancy of RoMPC with Moco does not necessarily reflect a low affinity and hence does not contradict the status of RoMCP as a Moco carrier protein. As Moco is not available in its free form inside the cell (Aguilar *et al.*, 1992 ▸), loading RoMCP with Moco would have to be achieved through a handshake with the ultimate protein of the Moco-biosynthesis pathway, *i.e.* the molybdenum insertase MoeA (Nichols & Rajagopalan, 2002 ▸; Sandu & Brandsch, 2002 ▸). A possible reason for the low occupancy of RoMCP with Moco could thus be poor compatibility with the *E. coli* Moco-biosynthesis machinery.

The low saturation levels measured in the Form A assay led us to speculate that RoMCP might favor the binding of MCD or MGD, which the standard Form A assay cannot detect. While the CrMCP reported by Fischer *et al.* (2006 ▸), which originates from a green alga and hence is unaccustomed to molybdenum dinucleotide cofactors, would preferentially bind Moco when expressed in *E. coli*, RoMCP could instead be selective for MCG or MGD. We put this notion to the test through *nit-1* reconstitution coupled to an NR activity assay, which is more sensitive than Form A detection but does not allow the quantification of Moco saturation levels. In this assay, RoMCP is able to transfer Moco to apo NR, hence reconstituting the NR activity, while the control protein is unable to do so (Table 5 ▸). As expected for such a transfer reaction, the reconstituted NR activity increases directly with the amount of Moco-loaded RoMCP added to the experiment but levels off at higher concentrations. Since NR depends on Moco and cannot utilize MCD or MGD, neither of the latter would contribute to the reconstitution of NR activity. Hence, we added phosphodiesterase (PDE) to the mixture to release Moco from its dinucleotide forms, allowing it to be transferred to apo NR. However, the reconstituted NR activity did not change significantly upon the addition of PDE, indicating that only Moco and neither MCD nor MGD is bound to RoMCP (Table 5 ▸). Considering that MCD and MGD represent the majority of the pool of Moco derivatives in the bacterial cell (Mendel & Leimkühler, 2015 ▸), this led us to conclude that RoMCP can discriminate between Moco and its dinucleotides, showing a selective affinity for the former.

### 3.4. Crystals of RoMCP and initial crystallographic analysis   

The crystals of RoMCP were typically colorless, except for color effects created by birefringence. The habit of the crystals was either plate-shaped or columnar, with the columnar crystals readily revealing the monoclinic crystal system (Fig. 2 ▸ *c*). The outer form agreed with the internal symmetry of the crystals, which was assigned to space group *P*2_1_, with unit-cell dimensions *a* = 68.58, *b* = 72.04, *c* = 70.26 Å and a monoclinic angle β = 113.33°, during data processing. Based on the empirical distribution of Matthews coefficients (Matthews, 1968 ▸), the most likely number of RoMCP molecules per asymmetric unit was four, corresponding to a Matthews coefficient of 2.21 Å^3^ Da^−1^.

### 3.5. The structure of RoMCP   

**Figure 3:**
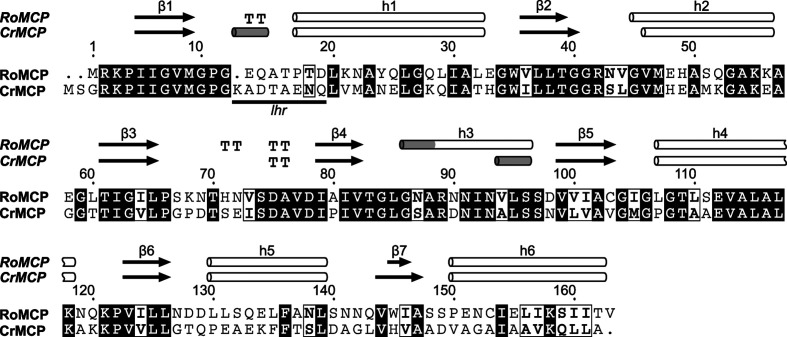
Sequence alignment of RoMCP with CrMCP. The respective secondary-structure elements are represented as cylinders and arrows above the sequence.

**Figure 4:**
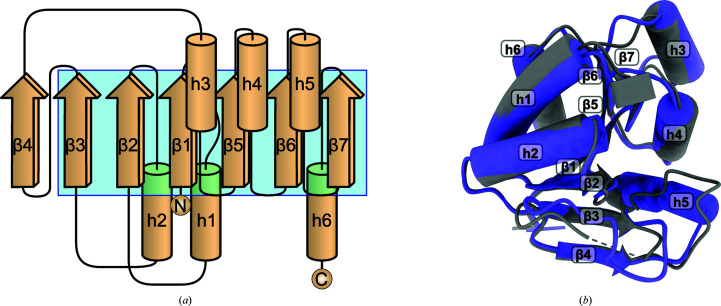
(*a*) Topology diagram of RoMCP. The cyan box indicates the core β-sheet of the canonical Rossmann fold. (*b*) Superposition of the RoMCP monomer (purple) with the CrMCP monomer (gray).

**Figure 5:**
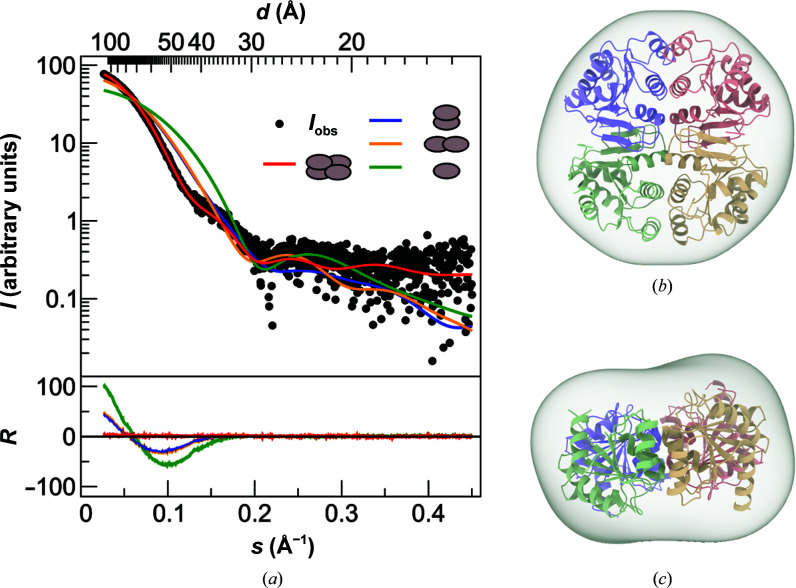
(*a*) Plot of SAXS intensities versus momentum transfer (on the *x* axis) and the corresponding resolution (on the alternate *x* axis) comparing experimental (*I*_obs_) with calculated intensities for the possible oligomeric assemblies. The residual *R* = (*I*_obs_ − *I*_obs_)/σ*I*_obs_ represents the discrepancy between the model selected from the crystal structure and the experimental data. (*b*) Front view and (*c*) side view of a SAXS envelope calculated from the experimental data with *DAMMIF* and superimposed with the tetramer *ABCD* from the crystal structure.

A monomer of the RoMCP construct in the crystal structure comprises 178 amino acids, 15 of which correspond to affinity and cloning tags. The electron density allowed the modeling of all amino acids of RoMCP in an uninterrupted polypeptide chain. In contrast, most amino acids of the affinity and cloning tags show disorder. An RoMCP monomer contains five α-helices (h1, h2, h4, h5 and h6) and one mixed helix (h3) that begins in a 3_10_ conformation and ends in an α conformation (see Fig. 3 ▸). The helices lie below (h1, h2 and h6) and above (h3, h4 and h5) a parallel β-sheet, resulting in a three-layered sandwich structure that resembles a Rossmann fold. In contrast to the canonical, six-stranded Rossmann fold, a seventh strand (denominated β4) expands the β-sheet of RoMCP, resulting in an arrangement with the order β4β3β2β1β5β6β7 (see Fig. 4 ▸). A nonregular stretch of amino acids between β1 and h1 contains the Rossmann consensus sequence G*x*G*xx*A/G that is usually involved in nucleotide binding. This stretch further comprises a type I β-turn motif. According to a sequence alignment of *mcp* gene products from different species, this stretch is part of a low-homology region (LHR) of variable length and amino-acid composition. A comparison of the tertiary structure of RoMCP with that of CrMCP reveals high similarity of the proteins: they superimpose with a C^α^ r.m.s.d. of 0.70 Å for the 140 best-aligning amino-acid pairs and of 1.48 Å for all pairs (Fig. 4 ▸ *b*). Only two conformational differences between the structures are notable. Firstly, in CrMCP the β-turn present in the LHR of RoMCP is replaced by a short 3_10_-helix. Secondly, the helix in CrMCP corresponding to the mixed h3 in RoMCP is shorter and does not contain α-helical elements. In the CrMCP structure, the content of the asymmetric unit comprises a dimer that is equivalent to the *AB* protomer pair in the RoMCP structure. However, CrMCP exhibits a tetrameric quaternary structure with 222 point-group symmetry generated from the content of the asymmetric unit by a twofold crystallographic rotation axis (Fischer *et al.*, 2006 ▸). This tetrameric assembly is identical to the content of the asymmetric unit in the RoMCP structure. Analysis of the RoMCP structure with the *Protein Interfaces, Surfaces and Assemblies *service *PISA* at the European Bioinformatics Institute (http://www.ebi.ac.uk/pdbe/prot_int/pistart.html; Krissinel & Henrick, 2007 ▸) confirmed the tetramer *ABCD* as well as the dimers *AB* and *AC* to be stable in solution. Comparison of theoretical small-angle X-ray scattering intensities calculated for the tetramer *ABCD*, the dimers *AB* and *AC* and the monomer *A* in the RoMCP structure with experimental intensities (*I*_obs_) collected from RoMCP in solution confirmed the tetramer to be stable and predominant at RoMCP concentrations as low as 38 µ*M* (Fig. 5 ▸), which rules out the tetramer *ABCD* being an artifact of crystallization. Within the tetramer, the interface of dimer *AB* comprises an area of 1023 Å^2^. It is formed by the three roughly antiparallel helix pairs ↑h3^*A*^↓h3^*B*^, ↑h4^*A*^↓h4^*B*^ and ↑h5^*A*^↓h5^*B*^ that consist of identical helices from both protomers. The interactions between protomers *A* and *B* are purely hydrophobic. In contrast, the interface between protomers *A* and *C* comprises an area of 875 Å^2^ and is formed by β-strands β3 and β4 interacting in a parallel orientation (↑β3^*A*^↑β4^*C*^ and ↑β4^*A*^↑β3^*C*^) through their hydrophobic side chains. A hydrogen bond between Val82 and Ser96 further stabilizes dimer *AC*, introducing a total of two hydrogen bonds to the dimer (Val82^*A*^ with Ser96^*C*^ and Val82^*C*^ with Ser96^A^). The involvement of strand β4 in this interface suggests that the expansion of the canonical Rossmann fold is essential for the stability of dimer *AC* and the tetramer. *PISA* estimated the solvation free-energy gain upon the formation of dimer *AB* to be 90.0 kJ mol^−1^, which is significantly greater than that for the formation of dimer *AC* (55.6 kJ mol^−1^), indicating that the formation of the RoMCP tetramer follows the route via a dimer *AB* intermediate.

**Figure 6:**
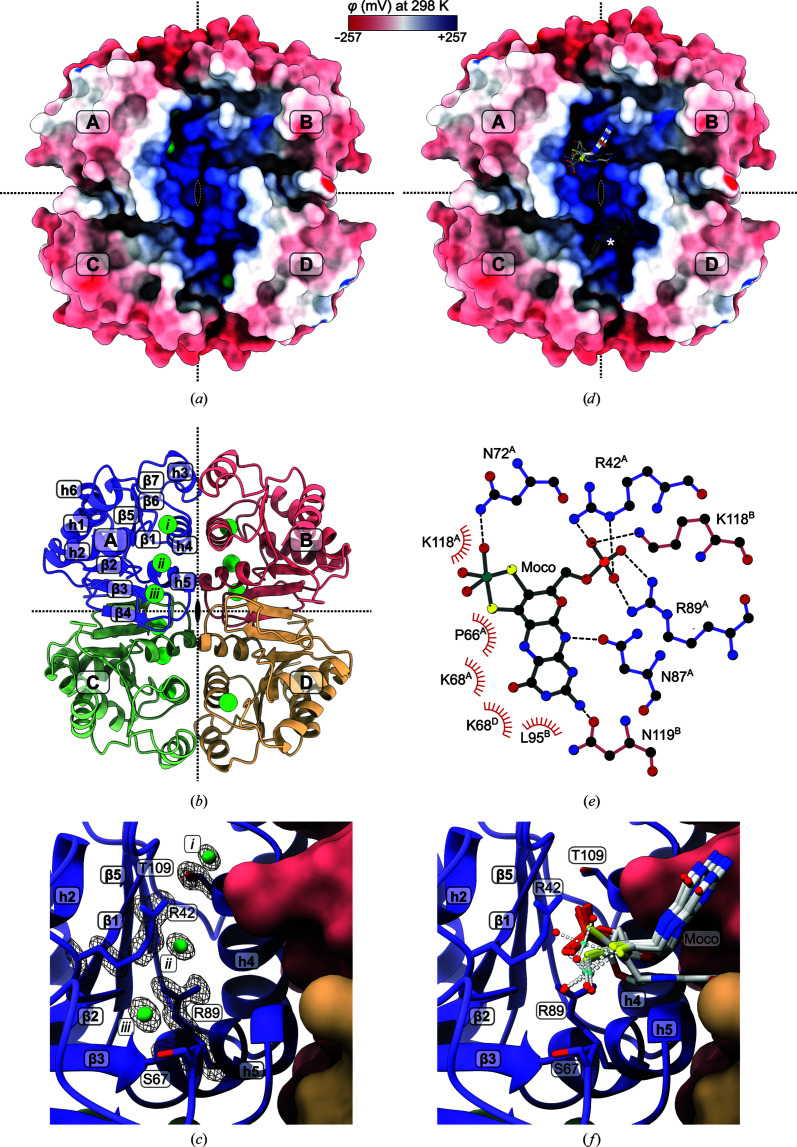
(*a*) The RoMCP 222 tetramer (dyads indicated as dotted lines and an ellipse) shown as a solvent-excluded surface with electrostatics. A positively charged crevice harbors up to three chloride anions. (*b*) The RoMCP tetramer in cartoon representation. (*c*) Close-up of the chloride anions in protomer *A*. (*d*) The five top-ranked results of the *GOLD* docking in protomer *A* (the equivalent binding site in protomer *D* is marked with an asterisk). (*e*) Schematic of interactions with the top docking result (hydrogen bonds and salt bridges are shown as dashed lines; hydrophobic interactions are shown as ‘eyelashes’). (*f*) Close-up of the putative Moco-binding site.

The surface of the RoMCP tetramer is predominantly negatively charged. However, a positively charged crevice forms at the front and back of the tetramer, which connects the diagonally opposed protomers *A* and *D* as well as protomers *B* and *C* (Fig. 6 ▸ *a*). In each protomer, up to three chloride anions reside within this crevice (*A*, 3; *B*, 3; *C*, 2; *D*, 1; Fig. 6 ▸ *b*) that are coordinated by Arg42, Arg89, Ser67 and Thr109 (Fig. 6 ▸ *c*). An analysis of homologous structures revealed that the equivalent positions frequently harbor anions such as sulfate or phosphate [see, for example, PDB entries 1t35 (New York SGX Research Center for Structural Genomics, unpublished work), 5wq3 (Seo & Kim, 2017 ▸) and 5zbj (Seo & Kim, 2018 ▸)]. Notably, some homologous structures of enzymes harbor the phosphate groups of substrates or catalytic products such as adenosine monophosphate and 5′-phosphoribose in this site [for example, PDB entry 5zbk, 24.8% sequence identity, 1.65 Å C^α^ r.m.s.d. with RoMCP (Seo & Kim, 2018 ▸) and PDB entry 5aju, 22.8% sequence identity, 1.83 Å C^α^ r.m.s.d. with RoMCP (Dzurová *et al.*, 2015 ▸)]. We thus reason that the positively charged crevice and the chloride anions bound within it mark the putative Moco-binding site of RoMCP.

### 3.6. The putative Moco-binding site   

We calculated 30 ranked docking models with *GOLD*: the top five models of this ranking form a cluster of positionally close molecules with identical orientations and very similar conformations. All Moco molecules in this cluster have their phosphate moiety in a position equal to that of chloride anion ii and their molybdenum center close to that of chloride anion iii in the original RoMCP crystal structure (Figs. 6 ▸ *d*, 6 ▸ *e* and 6 ▸ *f*). According to the docking, Moco binds near the interface of protomer *A* with protomers *B* and *D*, and maintains interprotomeric interactions. The electropositive amino acids Arg42^*A*^, Arg89^*A*^ and Lys118^*B*^ probably shield the negative charge of the phosphate group and hence contribute significantly to the net free-energy gain of Moco binding. Surprisingly, the negatively charged molybdenum center does not seem to be involved in ionic interactions but forms a hydrogen bond to Asn72^*A*^ in four of the top five docking models. Further contributions to Moco binding are likely to comprise hydrogen bonds between Asn87^*A*^ and the molybdopterin ring N atom N10 as well as between Asn119^*B*^ and the amino group attached to the ring atom C8. Hydrophobic interactions are provided mostly by protomer *A*, but to a smaller extent also by protomers *B* and *D*. Despite its location near the interface, Moco is at a reasonable distance from the dyads that relate the protomers, allowing one Moco molecule to bind per protomer.

Interestingly, the phosphate moiety of the Moco molecule resides in a deeply buried position within RoMCP. In contrast, the molybdenum center, which is the part of Moco that is most sensitive to oxidation, is relatively solvent-exposed. However, the exposed position of the molybdenum center resembles the situation in the docking model reported by Fischer *et al.* (2006 ▸) and does not seem to be in conflict with the protection from oxidation offered by MCPs.

The docking of MGD and MCD was inconclusive. A single RoMCP protomer alone cannot accommodate these bulky Moco derivatives. Although the crevice formed by the diagonally opposed protomers (Fig. 6 ▸ *a*) is spacious enough for a Moco dinucleotide, such binding would unevenly occupy two equivalent binding sites with the molybdopterin and the guanine/cytosine moiety, respectively, and thus break the 222 symmetry of the RoMCP tetramer. The docking hence supports the notion of RoMCP binding Moco.

## 4. Conclusions   

In the present work, we identified and characterized the first confirmed Moco carrier protein originating from a prokaryotic organism, the cyanobacterium *R. orientalis*. We showed that RoMCP selectively binds Moco and discriminates it from its dinucleotide derivatives. However, the saturation of RoMCP with Moco was low when expressed and purified from *E. coli*, probably owing to incompatibilities between the *E. coli* molybdenum insertase MoeA and RoMCP. We cannot exclude, however, that further tweaking of the expression and purification protocols could significantly increase the amount of Moco that can be co-purified with recombinant RoMCP.

Since our control experiments ruled out a nonspecific co-purification of Moco with RoMCP, we located the probable Moco-binding site and proposed a binding mode of Moco based on docking calculations, which agree with previously published docking studies. The identification of this RoMCP from an oxygenic bacterium underlines the necessity to protect Moco against oxidative damage in oxygen-evolving organisms.

## Supplementary Material

PDB reference: Moco carrier protein, 6y01

## Figures and Tables

**Table 1:** Macromolecule-production information

Source organism	*R. orientalis*
DNA source	Synthesized cDNA
Forward primer†	5′-atcGGATCCatgcgtaagccgattatcggcgttatg-3′
Reverse primer†	5′-gctgatcaagagcatcattaccgtcAAGCTTctg-3′
Cloning vector	pEX-A2
Expression vector	pQE80
Expression host	*E. coli* BL21
Complete amino-acid sequence of the construct produced‡	MRGSHHHHHHGSMRKPIIGVMGPGEQATPTDLKNAYQLGQLIALEGWVLLTGGRNVGVMEHASQGAKKAEGLTIGILPSKNTHNVSDAVDIAIVTGLGNARNNINVLSSDVVIACGIGLGTLSEVALALKNQKPVILLNDDLLSQELFANLSNNQVWIASSPENCIELIKSIITVKLN

† Restriction sites are shown in capitals.

‡ Affinity and cloning tags are underlined.

**Table 2:** Crystallization conditions

Method	Sitting-drop vapor diffiusion
Plate type	96-well plate
Temperature (K)	293
Protein concentration (g l^−1^)	22.5
Buffer composition of protein solution	0.025 *M* Tris–HCl pH 8.0, 0.3 *M* NaCl, 2%(*v*/*v*) glycerol
Composition of reservoir solution	20%(*v*/*v*) MPD, 0.1 *M* sodium acetate pH 5.0
Volume of drop (µl)	0.2
Ratio of drop	1:1
Volume of reservoir (µl)	60

**Table 3:** Data-collection and processing statistics

	Initial processing	After paired refinement
Diffraction source	X06DA, SLS	
Wavelength (Å)	1.000	
Temperature (K)	100	
Detector	PILATUS 2M-F	
Crystal-to-detector distance (mm)	120	
Rotation range per image (°)	0.1	
Total rotation range (°)	360	
Exposure per image (s)	0.1	
Space group	*P*2_1_	
*a*, *b*, *c* (Å)	68.58, 72.04, 70.26	
α, β, γ (°)	90, 113.33, 90	
Mosaicity (°)	0.15	
Overall Wilson *B* factor (Å^2^)	17.31	15.66
Resolution range (Å)	72.04–1.43 (1.45–1.43)	72.04–1.23 (1.25–1.23)
No. of reflections		
Total	794520 (39967)	1213870 (56141)
Unique	115894 (5732)	181767 (8965)
Completeness (%)	100 (100)	100 (100)
Multiplicity	6.9 (7.0)	6.7 (6.3)
〈*I*/σ(*I*)〉	18.3 (2.0)	12.0 (0.5)
CC_1/2_	1.000 (0.736)	1.000 (0.186)
*R*_meas_	0.049 (1.029)	0.066 (3.884)

**Table 4:** Structure solution and refinement

Resolution range (Å)	64.52–1.23 (1.27–1.23)
No. of reflections	
Working set	172800 (17241)
Test set	8865 (863)
Final *R*_cryst_	0.1633 (0.3656)
Final *R*_free_	0.1830 (0.3630)
No. of non-H atoms	
Protein	4835
Ion	10
Ligand	10
Water	393
Total	5248
R.m.s. deviations	
Bond lengths (Å)	0.018
Angles (°)	1.76
Average *B* factors (Å^2^)	
Protein	30.14
Ion	33.48
Ligand	85.57
Water	43.42
Ramachandran plot	
Most favored (%)	99.08
Allowed (%)	0.92

**Table 5:** NR activity in a *nit-1* extract reconstituted by recombinant RoMCP Sample size *N* = 4.

Amount (pmol)		NR activity (pmol min^−1^)		
Protein	Moco	−PDE	+PDE	*p*-value
7	0.03	4.94 (192)	2.73 (69)	0.13
14	0.06	4.61 (184)	2.99 (99)	0.25
27	0.12	5.16 (194)	4.07 (72)	0.41
54	0.23	7.43 (222)	5.48 (61)	0.21
109	0.47	11.29 (269)	7.63 (92)	0.09
217	0.93	19.60 (569)	10.39 (117)	0.52
434	1.87	22.31 (890)	13.07 (125)	0.15
868	3.73	18.90 (542)	16.28 (102)	0.45
1736	7.47	19.29 (606)	22.49 (212)	0.43
2901	12.53	19.80 (563)	30.27 (184)	0.04
Control: 1902	n.d.	n.d.	n.d.	—
